# Detection of *thyroglobulin, TSH receptor and sodium-iodine symporter* mRNA for identification of lymph node metastases in patients with papillary thyroid carcer

**DOI:** 10.1186/s12885-026-16515-z

**Published:** 2026-07-09

**Authors:** Lei Gao, Yu Bao, Tie-Xin Li, Guo-Qing Liu, Fu-Xing Zhang, Ying-Chao Chen, Bing Han, Lu Li, Huai-Dong Song, Mu-Lin Liu, Mei Dong

**Affiliations:** 1 Bengbu Medical University, Bengbu Anhui, 233030 China; 2 Department of General Surgery, The Central Laboratory of the First Affiliated Hospital of Bengbu Medical University, Bengbu Anhui, 233000 China; 3 Institute and Department of Endocrinology and Metabolism, Shanghai Ninth People’s Hospital, Shanghai Jiao Tong University School of Medicine, Shanghai, 200011 China; 4 Department of Endocrinology and Metabolism, Shanghai Medical College, Huashan Hospital, Fudan University, Shanghai, 200040 China; 5 Department of Molecular Diagnostics & Endocrinology, The Core Laboratory in Medical Center of Clinical Research, Shanghai Ninth People’s Hospital, State Key Laboratory of Medical Genomics, Shanghai Jiao Tong University School of Medicine, Shanghai, 200011 China

**Keywords:** Papillary thyroid cancer, Thyroglobulin, TSH receptor, Sodium-iodide symporter, Lymph node metastases, mRNA expression, qPCR

## Abstract

**Background:**

Papillary thyroid carcer (PTC) is the most prevalent endocrine malignancy, with the morbidity increase in recent years. The presence of metastatic lymph nodes stands as a significant predictor for recurrent occurrences of the disease. In this study, we explored the utility of biomarkers in aiding the identification of lymph node metastases in patients with PTC.

**Methods:**

We gathered 388 formalin-fixed paraffin embedded (FFPE) lymph node specimens from 266 individuals who had undergone surgical procedures for PTC. These samples were analyzed for the mRNA expression level of *thyroglobulin* (*TG*), *TSH receptor* (*TSHR*), and *sodium-iodide symporter* (*NIS*) using the real-time fluorescence quantitative PCR (qPCR) method. The results were compared with pathologic diagnosis.

**Results:**

Among 134 confirmed lymph node metastasis tissues, it was found that 123 samples expressed *TG* mRNA, 118 samples expressed *TSHR* mRNA and only 16 samples expressed *NIS* mRNA. Among 254 nonmetastatic lymph node tissues, 240 samples did not express *TG* mRNA, 219 samples did not express *TSHR* mRNA and 230 samples did not express *NIS* mRNA. The findings suggest that the measure of *TG* mRNA for the diagnosis of lymph node metastasis has a 91.79% sensitivity rate and a 94.49% specificity rate. The detection of *TSHR* mRNA displays an 88.06% sensitivity and an 86.22% specificity for recognizing lymph node metastasis. However, mRNA expression of *NIS* was not detected in most of lymph node samples.

**Conclusions:**

The research suggests that identifying *TG* mRNA expression in lymph nodes could serve as an effective approach to aid in postoperative diagnosing lymph node metastasis in patients with PTC.

## Background

Among the frequently occurring cancers of the endocrine system, thyroid carcer (TC) stands out, with its cases notably escalating in the past few years. Within this group, PTC emerges as the dominant type of TC [1, 2]. PTC is also the most prevalent TC subtype in countries having iodine sufficient or iodine-excess diets [3]. The reasons are unclear but may reflect improvements of methods for earlier detection of the cancer [4]. Typically, the onset of PTC occurs between the ages of 30 and 50, averaging around 40 years old. As individuals grow older, the likelihood of developing PTC rises, and it predominantly impacts females more than males, with gender disparities ranging from 2 to 1 up to 4 to 1 [4, 5]. Management of PTC typically comprises operative intervention and therapy using iodine 131 radioisotope, along with suppressive therapy with thyroid hormones. Ordinarily, patients with PTC have a better prognosis, with the 10-year survival rate exceeding 90%. Nevertheless, a quarter of individuals with PTC experience a relapse over an extended period of monitoring, potentially resulting in therapeutic resistance and mortality [6, 7]. Evidence suggests a significant link between lymph node metastasis and the recurrence of PTC [7, 8], highlighting the critical need for prompt and precise detection of lymph node metastasis to improve patient outcomes.

Post-surgical tissue analysis is regarded as the definitive method for detecting lymphatic metastasis in patients with PTC. Nonetheless, the accuracy of this diagnostic tool is contingent upon the expertise of the examining pathologist, and lesions of metastasis under 2 mm in size risk being overlooked based on the guidelines presently endorsed by the College of American Pathologists (CAP) and the American Society of Clinical Oncology (ASCO) [9]. The technique of measuring TG protein levels in neck lymph node fine needle aspiration washout fluid (FNA-Tg) as a dependable approach for identifying lymph node metastases in individuals diagnosed with PTC has gained considerable acceptance due to its notable precision and discernment [10]. Nonetheless, the cutoff value of FNA-Tg that would reliably diagnose lymph node metastasis remains undetermined, with varying degrees of sensitivity and specificity reported in different studies [11, 12]. In fact, when cutoff value of FNA-Tg ≥ 20 ng/mL was used to detect the lymph node metastasis, the specificity only achieved 66.7% [13]. So it is tempting to investigate alternative diagnostic techniques for lymph node metastases. In PTC, the neoplastic cells originate from follicular cells of the thyroid and typically exhibit a high degree of differentiation. It was reported that *TG* and *NIS* are expressed in the PTCs [14]. Furthermore, the utilization of immunohistochemical (IHC) techniques to identify thyroglobulin (Tg) aids in confirming or ruling out a TC cells source for metastases with an unidentified primary site [15]. Consequently, it is reasonable to speculate that detection of some gene mRNA expression level in the cervical lymph nodes would be an innovative approach for identifying lymph node metastasis in patients with PTC, given that the metastatic PTCs may continue to express certain genes characteristically expressed in norma thyroid cells [16].

This research investigated the relevance of *TG*,* TSHR*, and *NIS* mRNA expression levels with lymph node metastasis in postoperative formalin fixed lymph nodes surgically resected from patients with PTC.

## Materials and methods

### Participants

Three hundred eighty-eight FFPE lymph node samples from 266 patients diagnosed with PTC were collected in this study. The patients were undergone surgery at Bengbu Medical University’s First Affiliated Hospital during the period from January 2018 to July 2022. We set specific exclusion parameters, including (1) any prior neck injury (2) other concurrent or previous cancers; (3) a postoperative pathological diagnosis of another subtype of PTC or other pathological type; and (4) lack of complete and available postoperative pathology. Research procedures were in accordance with the Helsinki Declaration and received the endorsement of the Shanghai Ninth People’s Hospital’s Research Ethics Committee, with all participants providing their informed agreement. All participants have given their informed consent.

### RNA extraction

The RNAprep Pure FFPE kit (DP439, Tiangen Biotechnology Co., Ltd., Beijing) was employed to isolate RNA from FFPE tissues after making slight adjustments to the protocol. Paraffin slice samples with a thickness of approximately 15 microns are used to extract RNA. Initially, xylene was used to remove paraffin, and then ethanol at a concentration of 100% was applied for rehydration. Subsequently, the samples underwent proteolytic digestion using proteinase K, with a subsequent one-hour incubation period at 55℃. This was then extended by an additional quarter of an hour at a temperature of 80℃. Quantification was performed using NanoDrop (Thermo Scientific, Inc., Waltham, MA, USA). RNA was stored at -80 ℃ if the downstream experiments were not done immediately.

### Detection of *TG*, *TSHR* and *NIS* expression using qPCR

One hundred ng of total RNA was used as the template for the reverse transcription cDNA, using random hexamers and oligo dT primers according to the manufacturer’s instructions (Takara, RR047B). The Premix Ex Taq™ (Probe qPCR) kit (Takara, RR390A) was used for the quantitative polymerase chain reaction (qPCR) analysis on the QuantStudio 6 Flex Real-Time PCR System (ABI). The detailed protocols of the reverse transcription cDNA and qPCR were listed in Table 1. The *TG*, *TSHR* and *NIS* probes were labeled with 5’6-FAM and 3’MBG, while the housekeeping gene *GAPDH* probe was labeled with 5’VIC and 3’MBG. Table 2 listed the primers and probes utilized, with *GAPDH* serving as the control. *GAPDH* CT values within the range of 15 to 30 were indicative of qualified samples.

**Table 1:** Detailed protocols for reverse transcription cDNA and qPCR

procedure for reverse transcription cDNA			procedure for qPCR		
Step1 Mixture	reagents	volume	reagents		volume
	5XgDNA Eraser Buffer	2.0ul	2X Premix Ex Taq		10 ul
	gDNA Eraser	1.0ul	PCR Forward Primer (10 μm)		0.4 ul
	Total RNA	X ul(100ng)	PCR Reverse Primer (10 μm)		0.4 ul
	RNase Free dH_2_O	up to 10.0ul	Probe		0.8 ul
program	42℃ 2 min		ROX Reference Dye (50X)		0.4 ul
	85℃ 5s		cDNA template		1ul
Step2 Mixture	Step1 Mixture	10.0ul	RNase Free dH2O		7ul
	PrimeScript RT Enzyme Mix 1	1.0 ul	Total		20 ul
	RT Primer Mix	4.0 ul	program		
	5*PrimerScript Buffer 2	4.0 ul	step1	95℃ 2 min	one cycle
	RNase Free dH_2_O	1.0 ul	step2	95℃ 30 s	10 cycles without signal acquisition
	Total	20.0 ul		60℃ 30 s	
program	37℃ 15 min,		step3	95℃ 30 s	40 cycles with signal acquisition
	85℃ 5 s and 4℃ stock			64℃ 34 s	

**Table 2:** Primers used in the paper

GAPDH-F/*R*	5’-CTCAAGGGCATCCTGGGCTA-3’; 5’-CCAGCGTCAAAGGTGGAGGA-3’
TG-F/R	5’-CACGGATGACTATGCCTCCTT-3’; 5’-GGTACATGAAGACGTTTCCTCG-3’
TSHR-F/R	5’-GCTGGAATCACACTCCTTCTAC-3’; 5’-AGGGCATCAGGGTCTATGTA-3’
NIS-F/R	5’-CATCAGCTGCCTGACAGGC-3’; 5’-ACCAAGTTGTCATCCAGGATGG-3’
GAPDH probe	5`VIC-CACTGAGCACCAGGTGGTCTCCT-3`MGB
TG probe	5`6-FAM-AATGCCACCCGGGACTACTTTATCATC-3`MGB
TSHR probe	5`6-FAM-AGTGACTCACATAGAAATTCGGAATACCAGG3`MGB
NIS probe	5`6-FAM-CCTCGCACGGCAGACAGCATCA-3`MGB

### Statistical analyses

Data were statistically analyzed through the use of GraphPad Prism 10.0 software (GraphPad software, Boston, USA). The basic data of patients were compared by employing the Mann-Whitney U tests and the chi-square test. Significance was assigned to P ≤ 0.05. To ascertain the cutoff value for *TG*,* TSHR*, and *NIS* qPCR CT values in assessing metastatic lymph nodes, analysis of the receiver operating characteristic (ROC) curves was undertaken. For those which no CT value was detected we assigned them a value of 40 when analyzing ROC. The optimal cutoff value was established by identifying the peak value of Youden’s index. The ‘Area’ (representing the AUC value of the curve) was set as the standard for measuring efficacy, with a scale between 0.5 and 1, where values nearing 1 were deemed to indicate the highest success.

## Results

### Clinical characteristics of patients

The study cohort consisted of 266 patients with PTC. Among them, 197 individuals, or 74%, identified as female, while 69, representing 26%, were male. The patient’s mean age was at 46, with ages spanning from 12 to 75. From the cohort of 266 patients with PTC, 388 lymph node samples embedded in paraffin were collected, with the average number of resected lymph nodes being 1.46 per patients. Histopathological analysis after the initial surgery confirmed that 154 of these samples contained metastatic lymph nodes. The clinical and pathological information were provided in the Table 3. The average tumor size for men and women was 1.1 ± 1 cm and 1.1 ± 0.9 cm, respectively. There were 77 patients (22 male) with bilateral thyroid lesions, and 189 (47 male) with unilateral thyroid lesions, showing no significant difference in sex distribution (*p* = 0.53). The paraffin samples of lymph nodes with metastasis or without metastasis were recruited from the same patient with PTC for further research.

**Table 3:** The characteristics of the patients with papillary thyroid cancer

Variable	male(*n* = 69)	female(*n* = 197)	total(*n* = 266)	*p* value	statistical method
Age (Mean ± SD)(y)	45.9 ± 12.1	46.3 ± 12.1	46.2 ± 12.1	0.703	Mann-Whitney U test
Tumour diameter(cm)	1.1 ± 1	1.1 ± 0.9	1.106 ± 0.946	0.806	Mann-Whitney U test
≤ 1 cm	27	72	99	0.796	chi-square test
> 1 cm	42	125	167		
Bilateral lesions	22	55	77	0.532	chi-square test
Unilateral lesion	47	142	189		

### Evaluating the effectiveness of *TG*, *THSR*, and *NIS* mRNA expression level in identifying lymph node metastasis in patients with PTC

**Fig. 1:**
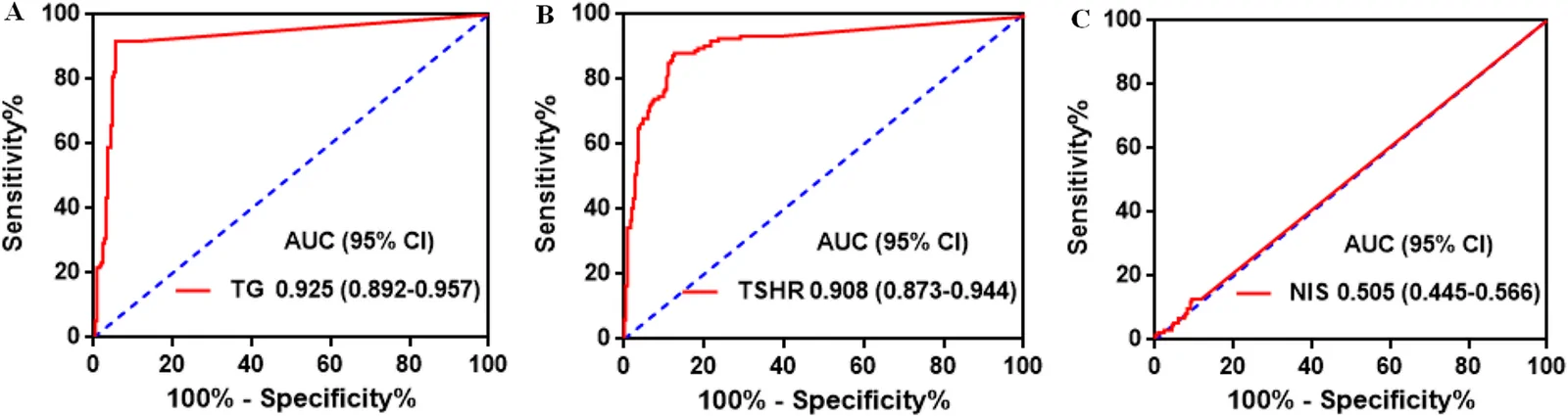
ROC curves for analysis cutoff of CT value of qPCR results for *TG*, *TSHR* and *NIS*. The area beneath the ROC curve is denoted as AUC. AUC was set as the standard for measuring efficacy, with a scale between 0.5 and 1, where values nearing 1 were deemed to indicate the highest success. ROC: the receiver operating characteristic.

This study was a retrospective study and the slides from the PTC patients’ lymph nodes were re-read by two histopathology expertise and these results were considered as the confirmed pathologic diagnosis. We detected the levels of *TG* mRNA in FFPE sample of the lymph nodes by qPCR, then compared the qPCR results with the confirmed histopathology. Utilizing the Receiver Operating Characteristic (ROC) graph, we ascertained the cutoff value for qPCR data. Selecting the cutoff value achieving the maximum Youden’s index, we pinpointed the optimal cutoff value of CT for *TG* in identifying lymphatic metastasis to be 29.98, accompanied by a Youden’s index of 0.87 (Fig. 1A). CT value above the cutoff was considered as mRNA expression negative.

Taken the confirmed postoperative pathologic diagnosis as gold in 134 metastatic lymph nodes, 123 exhibited *TG* mRNA ectopic expression, with the sensitivity 91.79%. While 240 out of 254 non-metastatic lymph nodes, *TG* mRNA expression was negative, with the specificity 94.49% (Table 4). The accuracy of the *TG* mRNA marker was measured at 93.56%, with the positive predictive value (PPV) and negative predictive value (NPV) achieving 89.78% and 95.62%, respectively, along with a Youden’s index of 0.87 (Table 4). It is important to mention that, there were no statistical difference between the results of *TG* mRNA and the first postoperative pathologic diagnosis. The first postoperative pathologic diagnosis showed a sensitivity of 96.27% which were greater than *TG* mRNA marker, yet it failed to achieve a significant statistical distinction (the first postoperative pathologic diagnosis vs. *TG* mRNA, 96.27% vs. 91.79%, *P* > 0.05) (Table 4). However, the specificity of *TG* mRNA detection was higher than that of the first postoperative pathologic diagnosis, but still did not reach the statistical difference (*TG* mRNA vs. the first postoperative pathologic diagnosis, 94.49% vs. 90.16%, *P* > 0.05) (Table 4). For other parameters, there were no statistical difference between the results of *TG* mRNA and the first postoperative pathologic diagnosis.(accuracy 93.56% vs. 92.27%, *P* > 0.05; PPV 89.78% vs. 83.77%, *P* > 0.05; NPV 95.62% vs. 97.86%, *P* > 0.05; and Youden’s index 0.87 vs. 0.87).

**Table 4:** Diagnostic performance of different modalities used in the correlation with confirmed pathologic diagnosis

Confirmed pathologic diagnosis	TG expression		TSHR expression		NIS expression		The first postoperative pathologic diagnosis	
	Positive	Negative	Positive	Negative	Positive	Negative	Metastasis	Non-Metastasis
Metastasis	123	11	118	16	16	118	129	5
Non-Metastasis	14	240	35	219	24	230	25	229
sensitivity	91.79%		88.06%		11.94%		96.27%	
specificity	94.49%		86.22%		90.55%		90.16%	
accuracy	93.56%		86.86%		63.40%		92.27%	
Positive Predict Value	89.78%		77.12%		40.00%		83.77%	
Negative Predict Value	95.62%		93.19%		66.09%		97.86%	
Youden index	0.87		0.74		0.02		0.87	

We then detected the *TSHR* and *NIS* expression using the same approach. By ROC analysis, the cutoff value of *THSR* and *NIS* were 27.18 and 39.32, respectively (Fig. 1B-C). Taken the confirmed pathologic diagnosis as gold standard, the *TSHR* mRNA marker’s performance in detecting metastatic lymph nodes in PTC patients showed a sensitivity of 88.06% (118/134), specificity of 86.22% (220/254). Compared with the *TG* mRNA marker and the first postoperative pathologic diagnosis, the sensitivity and specificity of *TSHR* mRNA marker were lower for diagnosing the metastatic lymph nodes in patients with PTC (sensitivity: *TSHR* vs. *TG*, 88.06% vs. 91.79%, *P* > 0.05, *TSHR* vs. the first postoperative pathologic diagnosis, 88.06%vs.96.27%, *P* < 0.05; specificity: *TSHR* vs.*TG*, 88.22% vs. 94.49%, *P* < 0.001, *TSHR* vs. the first postoperative pathologic diagnosis, 88.22% vs. 90.16%, *P* > 0.05). The *TSHR* mRNA detection showed accuracy of 86.86% (337/388), a PPV of 77.12% (118/153), and a NPV of 93.19% (219/235), with a Youden’s index of 0.74 as (Table 4). However, as most of the samples showed negative results for *NIS* mRNA detection, the sensitivity, specificity, accuracy, PPV and NPV of *NIS* mRNA marker for metastatic lymph nodes in patients with PTC were 11.94% (11/134) and 90.55% (235/254), 63.40% (246/388), 40% (16/40), and 66.09% (230/348), respectively, as well as Youden’s index 0.02 (Table 4).

## Discussion

PTC is characterized as high differentiation and considered as a condition with a minimal likelihood of recurrence, reaching a 99% survival rate two decades post-operatively [17]. However, a retrospective analysis of 269 PTC patients with 27 years of median follow-up revealed that 28% of recurrences and 9% of deaths [18]. Lymph node metastasis is the most common risk factor associated with the recurrence of PTC with an incidence rate of 20–50% [18, 19], and increased risk of death in PTC patients older than 45 [20, 21]. Thus, determining lymph node metastasis status is very important to improve prognosis and reduce mortality. However, evaluating the status of cervical lymph nodes is a big challenge, especially at the pre-operation. Ultrasound is one of the most common diagnostic methods for TC. However, the ultrasound is an operator-dependent tool, leading to variable accuracy [22]. FNA technology plays an increasingly important role in determining the status of lymph node metastasis. However, standard FNAB (fine-needle aspiration biopsy) is nondiagnostic in 25% to 40% of cases on average, which include inadequate specimens and indeterminate (suspicious) diagnoses [23].

Postoperative pathology is accepted as a gold standard to diagnose lymph node metastases in PTC patients. However, in the actual clinical diagnosis process, the pathological results depend on the professional skills of the pathologists and micrometastases (diameter < 2 mm) are under the detection threshold based on the guidelines endorsed by the College of American Pathologists (CAP) and the American Society of Clinical Oncology (ASCO) [9]. In our study, compared to the confirmed pathologic diagnosis, 25 lymph nodes originally diagnosed as metastatic, were considered non-metastatic by the two re-evaluaters. And 5 lymph nodes originally diagnosed as non-metastatic, were diagnosed as metastatic in the confirmed pathology, with the sensitivity, specificity and accuracy of the first pathological results of 96.27%, 90.16% and 92.27%, respectively. As reading pathology slices is highly dependent on the experience of the pathology experts, the discrepancy may be caused by the evaluaters, highlighting the necessity of developing other methods to assist the diagnosis.

Thyroglobulin (Tg) is a dimeric protein with a molecular mass of 660 kilodaltons, synthesized by thyroid follicle cells, playing a critical role in the production of thyroid hormones. Both non-malignant and highly differentiated cancerous thyroid cells secrete Tg [24, 25]. Developed over 30 years ago, measuring Tg levels in the fluid from fine needle aspirations (FNA-Tg) has become an important supplementary technique to identify lymph node metastases in patient with PTC [8]. However, the results were affected by a series of factors such as detection methods [26], reagent kits [27], anti-TG autoantibodies (TgAb) [28], and so on. Moreover, the cutoff value of FNA-Tg does not have a standard. According to a meta-analysis, the FNA-Tg cutoff values ranges from 0.2 to 50 ng/ml with different methods, with the sensitivity ranged from 69% to 100% and the specificity ranged from 25% to 100% [12].

Given that *TG*, *TSHR* and *NIS* are still expressed in papillary thyroid carcinoma cells [16], we presumed that measurement of the mRNA level in tissues of the cervical lymph nodes might be an alternative way to diagnose the cervical lymph node metastasis in patients with PTC.

In this retrospective study, the mRNA levels of *TG*,* THSR* or *NIS* were measured by qPCR in FFPE slices of 134 metastatic and 254 non-metastatic lymph nodes from 266 patients with PTC. In these markers only *TG* is exclusive expressed by the thyroid follicle cells. *TSHR* produces TSHR, a crucial membrane-bound protein that significantly influences thyroid cellular metabolic processes. Studies reported that besides thyroid follicular cells *TSHR* is expressed in immune cells as well [29–31].NIS localized in cytoplasmic membrane, facilitating the active uptake of iodide into the thyroid as well as other non-thyroid tissues [32]. Therefore, we assumed that detection of *TG* mRNA in lymph node would be a best method to help diagnose lymph node metastasis. Indeed, although the sensitivity of *THSR* was lower than TG (88.06% & 91.79%), there was no significant difference (*p* = 0.41). While there was significant difference of specificity between *THSR* and *TG* (86.22% & 94.49%, *p* = 0.024). The background expression of *TSHR* in certain types of immune cells may be the reason for its lower specificity compared to *TG* [29–31]. NIS-mediated radioactive iodine (RAI) therapy is the first-line treatment for patients with PTC. However, due to the lacking of *NIS* expression or NIS localization on plasma impairing, 60% of patients with aggressive metastasis PTC developed resistance to RAI treatment and had a poor overall prognosis [33]. We aimed to investigate *NIS* expression levels within the lymph nodes of patients with PTC, and wanted to correlate *NIS* expression level with RAI treatment effect in patients with PTC. However, we found that *NIS* expression was considerably less than that of *TG* and *TSHR*, to the extent that its mRNA expression was undetectable in the majority of our samples. It has been reported that although *NIS* was expressed in carcinomas but the expression level was much lower than in normal thyroid cells [14, 34]. In this study, only 100ng total RNA was used to reverse transcript cDNA, Therefore, the expression level of *NIS* may be below the detection threshold, resulting in detection negative.

The sensitivity of *TG* was 91.79%, which was lower than we expected. There were 11 lymph node samples that the pathologic diagnose was metastasis but the mRNA expression of TG was negative. Out of the 11 specimens, 8 were preserved during the period of 2018–2020, while the rest 3 specimens, were preserved in the subsequent years of 2021–2022. Consequently, the prolonged storage may have led to the deterioration of mRNA within these specimens. Conversely, the transition towards de-differentiation occurs incrementally, leading to the emergence of tumor cells capable of metastasis and the advancement of PTC towards a less differentiated and ultimately anaplastic form of TC [35, 36]. Extensive research from both laboratory experiments [37, 38] and patient studies [39] suggests that when PTC cells lose their specialized features, this process correlates with rapid tumor expansion, evidenced by a higher Ki-67 proliferation rate and reduced levels of thyroid-specific differentiation indicators, including *TG* and TTF-1. Besides technical factors, our presumption is that the undetectable *TG* expression observed in these individuals could stem from an increased severity of the cancerous cells and a greater potential for the tumor to recurrence. We intend to maintain ongoing observation over an extended period for patients with PTC to confirm our hypothesis. There were 14 samples in 254 lymph node (5.5%) that *TG* mRNA detection positive but pathologic diagnosis were negative. According the current approach recommended by both CAP and ASCO, metastasis lesions smaller than 2 mm may be missed in postoperative slides [9]. In these samples the metastasis lesion may be too small to be detected by pathology, highlighting the importance of biomarkers as a very good supplement to the existing diagnostic methods.

This study has several limitations. Firstly, the samples for *TG* mRNA measurement are postoperative lymph nodes, and the results cannot be used for preoperative assessment of lymph node metastasis status. FNA tissues will be used to measure *TG* mRNA expression level in the future. Secondly, although our results showed that *TG* mRNA detection has a good ability to predict lymph node metastasis, more samples and more clinical centres should be participated in to further validate the feasibility of the method.

## Conclusions

In this study, we investigated a new method for diagnosing lymph node metastasis in PTC patients. When compared with confirmed histopathology the results showed that *TG* and *TSHR* mRNA expression level could diagnose lymph node metastases with specificities and sensitivities of 91.79% and 94.49% for *TG*, and 88.06% and 86.22% for *TSHR*, respectively. But *NIS* was not detected in most of the lymph nodes, no matter benign or metastasis. The findings indicate that measuring *TG* mRNA expression level in FFPE lymph nodes could be an innovative approach for assisting the postoperative pathological diagnosis of lymph node metastasis in patients with PTC.

## Data Availability

Upon a justifiable inquiry, the corresponding author can provide the datasets that were utilized or examined throughout this study.

## Abbreviations

TG: Thyroglobulin. TSHR: TSH receptor
NIS: Sodium-iodide symporter
PTC: Papillary thyroid cancer
TC: Thyroid carcer
